# Cranial Nerve Foramina Part I: A Review of the Anatomy and Pathology of Cranial Nerve Foramina of the Anterior and Middle Fossa

**DOI:** 10.7759/cureus.2172

**Published:** 2018-02-08

**Authors:** Bryan Edwards, Joy MH Wang, Joe Iwanaga, Marios Loukas, R. Shane Tubbs

**Affiliations:** 1 Department of Anatomical Sciences, St. George's University School of Medicine, Grenada, West Indies; 2 Department of Anatomical Sciences, St. George's University School of Medicine, Grenada, West Indies; 3 Seattle Science Foundation; 4 Neurosurgery, Seattle Science Foundation

**Keywords:** foramina, cranial nerve, anterior fossa, middle fossa, cribiform plate, sphenoid, superior orbital fissure, optic canal, foramen rotundum, foramen ovale

## Abstract

Cranial nerve foramina are integral exits from the confines of the skull. Despite their significance in cranial nerve pathologies, there has been no comprehensive anatomical review of these structures. Owing to the extensive nature of this topic, Part I of our review, presented here, focuses on the foramina of the anterior and middle cranial fossae, discussing each foramen’s shape, orientation, size, surrounding structures, and structures that traverse them. Furthermore, by comparing the size of each foramen against the cross-sectional areas of its contents, we estimate the amount of free space in each. We also review lesions that can obstruct the foramina and discuss their clinical consequences.

## Introduction and background

Cranial nerve foramina are integral exits from the confines of the skull. On their long intracranial journeys and subsequent passage through these skeletal portals, cranial nerves can travel alone or with accompanying vascular structures. The foramina can sometimes become too small, or pathological obstructions (e.g., achondroplasia, fibrous dysplasia, osteopetrosis) can develop and impinge upon them, with potentially severe clinical consequences.

**Figure 1:**
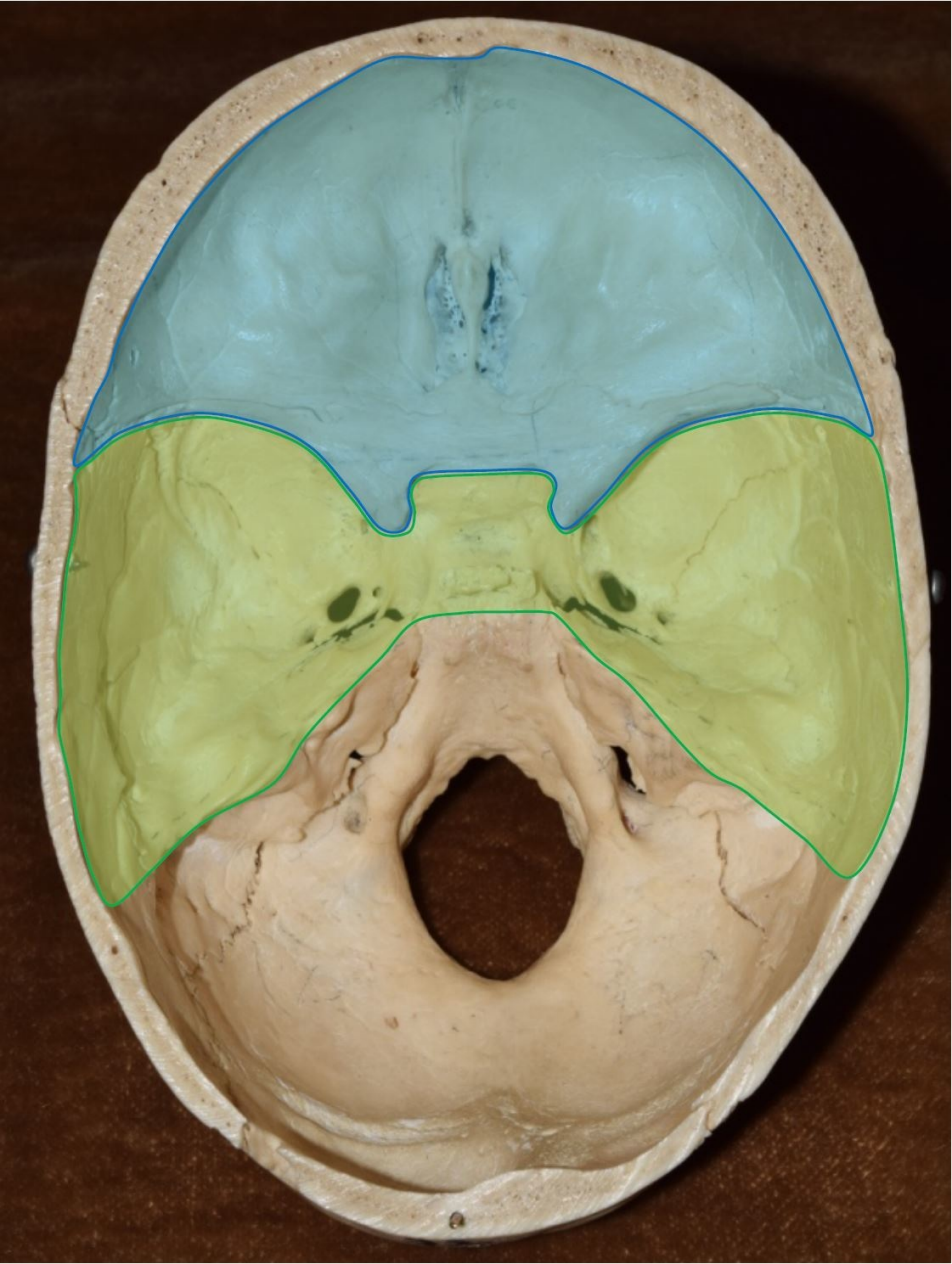
Superior view of cranial floor Blue - Anterior Cranial Fossa; Green - Middle Cranial Fossa

In this review, we describe the anatomy of the cranial nerve foramina of the anterior and middle fossa (highlighted in blue and green, respectively, in Figure 1) in terms of locations within the skull, shapes, dimensions, crucial surrounding structures, and any documented variations. The structures passing through these foramina and their corresponding sizes within them are reviewed by comparing their respective cross-sectional areas. Finally, pathological obstructions of the foramina and impingement of their contents are reviewed, along with the corresponding clinical consequences. To our knowledge, this is the first comprehensive review of the cranial nerve foramina of the anterior and middle fossae.

Limitations

Information regarding structural diameters, sizes of lesions, and measurements of masses extending into these foramina is seldom or never reported in the literature.

## Review

Middle cranial fossa

Foramen Ovale (FO)

**Figure 2:**
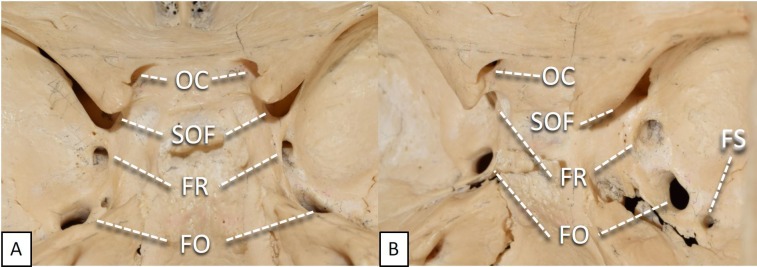
Close-up view of cranial nerve foramina within middle cranial fossa A: Superior view;  B: Oblique view. OC: optic canal; SOF: superior orbital fissure; FR: foramen rotundum; FO: foramen ovale; FS: foramen spinosum.

Situated in the posterior aspect of the lesser sphenoid wing and anteromedial to the sphenoid spine, the foramen ovale (Figure 2) adopts various shapes including oval, almond, round, and slit [1-2]. The predominant shape is oval, with dimensions ranging from 5 x 2 mm to 8 x 7 mm, the average being 7.11 x 3.60 mm [1, 3-4]. The bilateral comparison shows a slight asymmetry in the cross-sectional area, the right being 16.55 mm^2^ and the left 14.39 mm^2^. As with other foramina, ossification can divide it into two separate compartments; a full division has been reported in 2.8% of cases and a partial division in 12.8% [1]. Directly inferior to the exocranial surface of the FO, two ossified ligaments, known as the pterygospinous bar and the pterygoalar bar, are found in some cases. The prevalence of the pterygospinous bar, also known as the ligament of Civinini, has been reported as 2.6 - 17%, while that of the pterygoalar bar, also known as the ligament of Hyrtl, has been reported as 2.6 - 30% [5-6]. These bars can be unilateral and/or extend additionally over the foramen spinosum [7].

Surrounding this foramen are several important landmarks and structures. Medial to the FO, but lateral to the sella turcica, lies the cavernous sinus with its own important structures. Three millimeters posterolaterally lies the foramen spinosum, which contains the middle meningeal artery and the recurrent branch of the mandibular nerve. The carotid artery runs directly posterior to the intracranial opening of the foramen. The foramen rotundum lies 8 - 10 mm anteromedially and holds the maxillary branch of the trigeminal nerve [8]. Directly superior to it are the temporal lobes and the heart of the trigeminal nerve within Meckel’s cave [9]. Inferior to it is the intratemporal fossa. The posterior clinoid process is located 26.1 mm superolaterally.

The main structure running through the FO is the mandibular branch (V3) of the trigeminal nerve. Other structures include the accessory branch of the middle meningeal artery, the lesser petrosal nerve, small emissary veins, and the middle meningeal artery [7, 10]. The venous plexus, joining the cavernous sinus to the pterygoid plexus, can also run through this path [11]. If we compare the cross-sectional area of the mandibular nerve, 7.8 - 14.5 mm^2^ on the right and 10.4 - 16.2 mm^2^ on the left [12], with that of the foramen, 16.55 mm^2^ on the right and 14.39 mm^2^ on the left [7], it is clear that lesions can potentially obstruct the FO.

Extrinsic carcinomas appear to be the main cause of FO obstruction with clinical consequences. Laine et al. described three patients who had FO obstructions secondary to an adenoid cystic carcinoma traveling medially into the foramen [9]. Some of these patients presented only with the sensation of an enlarged mass on the side of their face, while others reported facial pain and displayed signs of masticator atrophy and cavernous sinus syndrome – a paresis of the oculomotor, trochlear, and abducens nerves. Obstruction by a squamous cell carcinoma has also been reported. Laine et al. reported several cases of FO obstruction secondary to squamous cell carcinoma, in which squamous cells from the maxillary sinus traveled deep into the skull via the trigeminal nerve, entering the FO, the superior orbital fissure, and the pterygoid fossa [9]. These patients experienced a combination of facial pain, chronic nasal discharge, and atrophied muscles of mastication. Barakos et al. reported a squamous cell carcinoma that spread from the lower facial region to the FO perineurally. The patient suffered progressive dysesthesia on the right side of the face along the distribution of the mandibular nerve [10]. Non-Hodgkins lymphoma originating from the orbit has also been shown to invade the FO and cavernous sinus; similarly, non-Hodgkins lymphoma originating from within the cranial cavity has been documented to extend through the FO along the mandibular branch [13].

The FO is therapeutically important as an access to Meckel’s cave in procedures, such as radiofrequency trigeminal rhizotomy and balloon compression, used to alleviate symptoms of trigeminal neuralgia. However, anatomical variations should be kept in mind, as Tubbs et al. reported cases of elongated or enlarged sphenoid spines that can obstruct and prevent glycerol rhizolysis [5]. Anatomical variations of the ligaments of Civinini and Hyrtle can also have clinical implications; if they compress the branches of the mandibular nerve, mastication weakness and sensory loss can result [6].

Foramen Rotundum (FR)

As the gateway for the maxillary branch of the trigeminal nerve, the foramen rotundum (Figure 2) sits vertically in the anteromedial portion of the greater sphenoid wing. It is posteroinferior to the medial edge of the superior orbital fissure, lateral to the anterior aspect of the sella turcica, inferior to the anterior clinoid process, and superior to the sphenoid bone and continuing nasal cavity. The foramen itself is circular with an average diameter of 3.9 x 3.13 mm [4], the left being slightly wider than the right [3]. In 1% of skulls, an accessory canal originates from the lateral wall of the FR and travels superiorly and diagonally to the orbit; its diameter and length have been measured as 0.2 - 1.0 mm and 5.0 mm, respectively [14-15]. The cross-sectional area of the FR is 9.48 mm^2^ and 9.45 mm^2^ on the right and left, respectively [16]. Interestingly, although the dimensions of the right and left sides are nearly identical, Neto et al. found a greater incidence of trigeminal neuralgia on the right [17]. In view of the wide-ranging cross-sectional area of the maxillary nerve (6.2 - 15.3 mm^2^ on the right and 4.6 - 15.3 mm^2^ on the left) [12], the amount of free space is highly variable so the fit can be tight, easily allowing a mass to impinge on the nerve within the foramen.

Hedeman et al. reported a case in which a malignant schwannoma in the heart of the trigeminal ganglion, also known as the Gasserian ganglion, protruded into the FR, causing right-sided pain and a burning sensation along the distribution of the maxillary nerve [18]. A 5 cm non-Hodgkins lymphoma originating from the parotid gland invaded the FR of a 17-month-old girl presenting with face and neck masses.

Superior Orbital Fissure (SOF)

Sitting in the sphenoid bone, the superior orbital fissure (Figure 2) comprises several parts: its superior and inferior borders are the lesser and greater wings of the sphenoid, respectively; its medial inferior edge is composed of the body of the sphenoid; and a small portion of the lateral aspect is contributed by the frontal bone. Superomedial to the SOF is the optic canal, separated by the optic strut; behind this structure runs the intracranial portion of the carotid artery. The FR, transmitting the maxillary branch of the trigeminal nerve, lies directly inferior to the medial base of the SOF. Posteriorly is the cavernous sinus, which lies lateral to the sella turcica. Attached to the medial side is the annular tendon, which gives rise to the extraocular muscles. The tendons of these muscles divide the fissure into three compartments: 1) the lateral sector (formed by the tendons of the lateral rectus and the lateral border of the SOF) containing the trochlear nerve, the frontal and lacrimal nerves (branches of V1), and the superior ophthalmic vein (listed lateral to medial); 2) the central sector (formed by the tendons of the inferior and lateral rectus) containing the oculomotor, nasocilliary (branch of V1), and abducens nerves and roots from the ciliary ganglion; and 3) the inferior sector (formed by the tendons of the inferior rectus and medial border of the SOF) containing the inferior ophthalmic vein, orbital fat, and smooth muscle [19].

The length of the fissure ranges from 20.05 - 22.0 mm [4, 20], with apical and basal widths of 2 - 3 mm and 7 - 8 mm, respectively [20]. Owing to its odd shape, the cross-sectional areas of the SOF and its passing structures have not been well documented. One study reported that the trochlear nerve has an average diameter of 0.54 mm [21]. Using the formula for the area of a circle, we calculate the cross-sectional area of the trochlear nerve to be 0.23 mm^2^. Clinically, obstructions at the SOF can have significant detrimental effects on vision.

A condition known as SOF syndrome (SOFS) is caused by the compression or obstruction of the structures passing through the SOF; it typically presents with a mixture of signs and symptoms, including ipsilateral unreactive pupil, ptosis, proptosis, ophthalmoplegia, and anesthesia of the upper eyelid and forehead [22-23]. Trauma and fractures appear to be the culprits in most of these cases. Rai et al. and Evans et al. reported several cases of direct fractures of the lateral wall of the orbit and indirect compression of the SOF secondary to zygomaticomaxillary fractures [20, 22]. They resulted in ophthalmoplegia, ptosis, proptosis, numbness over the forehead, dilated pupils, loss of corneal and direct light reflex, while the consensual light reflex remained intact, and loss of accommodation with no evidence of optic nerve injury [20]. The lateral wall of the orbit can also be affected by a frontosphenotemporal fracture, leading to impingement on the structures within the SOF [24]. Carcinoma can also cause SOFS. Kleydman et al. reported a case in which a basal cell carcinoma of the left nasal ala retrogradely invaded the orbit, including the SOF and much of the skull base. Because most of the involvement was around the orbit, the patient experienced bilateral vision loss, tearing, and swelling [25]. Squamous cell carcinomas have also been reported [26]. Infection can also cause compression of these structures. Aspergillus fumigates extending superiorly from the right sphenoid sinus was the reported cause of SOFS in a 60-year-old man presenting with ptosis, ophthalmoplegia, and forehead hypoesthesia [27]. Chronic tertiary syphilis can also have this effect [28].

Optic Canal (OC)

Allowing passage for the messenger of sight, the optic canal sits in the superior aspect of the sphenoid body and directly transverses outward into the medial wall of the orbit (Figure 2). Directly anterior to the tip of the anterior clinoid process and slightly anteromedial to the sella turcica, the optic canal is the most superiorly situated foramen in the middle fossa. It is anterior to the cavernous sinus, superolateral to the sphenoid sinus, and inferior to the frontal lobes. Anteromedial to the OC is the ethmoid bone. The triangular area that contains the foraminal openings of the optic canal, along with the ethmoid bone, is called the planum sphenoidale. The prechiasmatic sulcus is the groove on the anterior aspect of the sphenoid body connecting the openings of the two canals. The distal end of the OC is narrower than the proximal end [29]; the intracranial and orbital openings having average dimensions of 6.25 x 3.70 mm and 4.75 x 5.46 mm, respectively [4]. The canal itself is 8 - 12 mm long and is duplicated in 2.57% of skulls [30].

The optic nerve, with a cross-sectional area of 5.17 mm^2^ [31], runs through the foramen beside the ophthalmic artery. This artery has a diameter of 0.7 - 1.8 mm [32]; using the formula for the area of a circle, we find that its cross-sectional area is 0.38 mm^2^ to 2.54 mm^2^. Summing the areas, we obtain 5.55 to 6.97 mm^2^. Comparing this with the calculated cross-sectional areas of the orbital and intracranial openings, 18.16 mm^2^ and 20.33 mm^2^, respectively, we find 11 mm^2^ of free space. Therefore, a lesion around 1 cm would be expected to compress the internal structures. As Jackson et al. remarked, small tumors in the canal can produce symptoms but can be difficult to see [33].

Meningiomas, the most common intracranial tumors, involve the orbit in less than 2% of cases. However, when they do invade the canal, they cause progressive vision loss [34]. The usual patient presentation is a female between 24 and 38 years of age presenting with cloudy vision, decreased visual acuity, loss of color vision, or complete loss of vision, with possible findings of papilledema and pallor discs secondary to ophthalmic artery compression [33, 35]. From the planus sphenoidale, meningotheliomatous meningiomas could also extend posterolaterally into the canals [34]. Meningiomas extending from the tuberculum sella have been shown to invade the OC in 77.4% of cases, causing vision loss in 84.6% of those cases [36]. From within the canal itself, the ophthalmic artery can compress the optic nerve. A fusiform, irregular aneurysm of the ophthalmic artery, measuring approximately 4 x 3 mm and running the entire length of the canal, was reported in a 53-year-old man presenting with a progressive decrease in visual acuity and color perception [37]. The cross-sectional area of this aneurysm, using the formula for the area of an ellipse, would be 9.41 mm^2^, which is considerable since less than 11 mm^2^ of free space is available within the canal. A mucocele involving the left anterior clinoid process and optic strut was reported to compress the optic nerve in the left OC causing headaches, vision loss, and amaurotic pupil with mild impairment of abduction and corneal sensation. Fracture of the lesser wing of the sphenoid and posterior aspect of the ethmoid bone reportedly causes complete infarction of the ophthalmic artery in the canal, either from direct rupture of the artery secondary to the force of the trauma or from indirect compressive forces secondary to subsequent swelling and edema [38]. Fibrous dysplasia can also lead to optic nerve compression. Of the 93 patients studied, Tan et al. reported that 18 had evidence of OC involvement, 14 of whom suffered symptoms of complete blindness, loss of certain visual fields, or loss of color vision [35]. Additionally, osteopetrosis has been associated with stenosis of the optic canal with optic nerve atrophy [39].

Anterior compartment

Foramina of the Cribriform Plate

**Figure 3:**
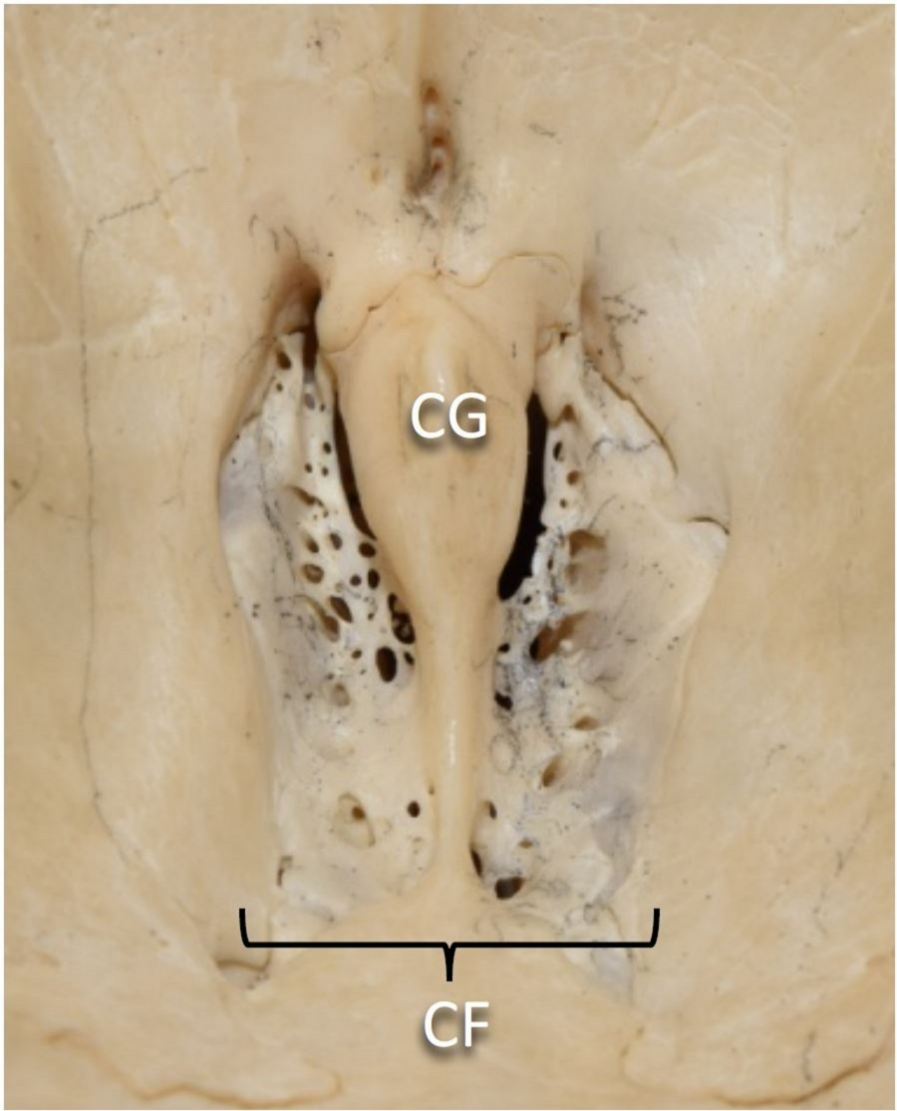
Close-up view of cranial nerve foramina within anterior cranial fossa CG: crista galli; CF: cribriform plate

The cribriform plate is the gateway to the nasal passages from inside the skull (Figure 3). It is interposed between the frontal and sphenoid bones. It lies horizontally with multiple foramina less than 1 mm in diameter perforating through it [40]. The olfactory bulb sits directly superior to it, transmitting the olfactory nerves from the nasal mucosa below. The total area of the perforations is age-dependent; it is 3.79 - 3.99 mm^2^ in those over 50 years old and 5.61 - 7.91 mm^2^ in those under 50. This decrease in the area over time, causing compression and dysfunction of the olfactory nerves, is thought to explain the decreased olfactory sensation in the elderly [41].

Clinically, most lesions affecting the foramina of the cribriform plate are schwannomas and meningiomas near the olfactory groove, causing headaches, seizures, personality changes (due to the proximity to the frontal lobes), and visual field defects (due to the proximity of the optic chiasm) [42-45]. Interestingly, anosmia is typically not a complaint and is only noted in hindsight [44]. This could be attributed to the slow rate of growth of these tumors.

## Conclusions

This review has examined the cranial nerve foramina of the anterior and middle fossae. When their dimensions are compared with the size of their contents, we can estimate the free space available; it is evident that lesions do not have to be large to cause impingement and significant clinical consequences. Understanding of these osseous defects is important for many disciplines in clinical medicine and surgery. We hope this review elucidates the spatial anatomy and various pathologies of the anterior and middle fossa foramina.
